# Supplementary material for: Am80‐GCSF synergizes myeloid expansion and differentiation to generate functional neutrophils that reduce neutropenia‐associated infection and mortality
Source: EMBO Mol Med. 2016 Oct 13;8(11):1340–59. doi: 10.15252/emmm.201606434 (PMC5090663; doi:10.15252/emmm.201606434)
Supplement: Supplementary file 1 — Appendix [file EMMM-8-1340-s001.pdf]

Appendix

1. **Appendix figures and figure legends S1 to S5……………………..Page 2-8**
2. **Appendix tables and table legends S1 to S4………………………..Page 9-24**
3. **References…………………………………………………………..Page 25**

1) Appendix Supplemental Figures and Figure Legends

Appendix Figure S1

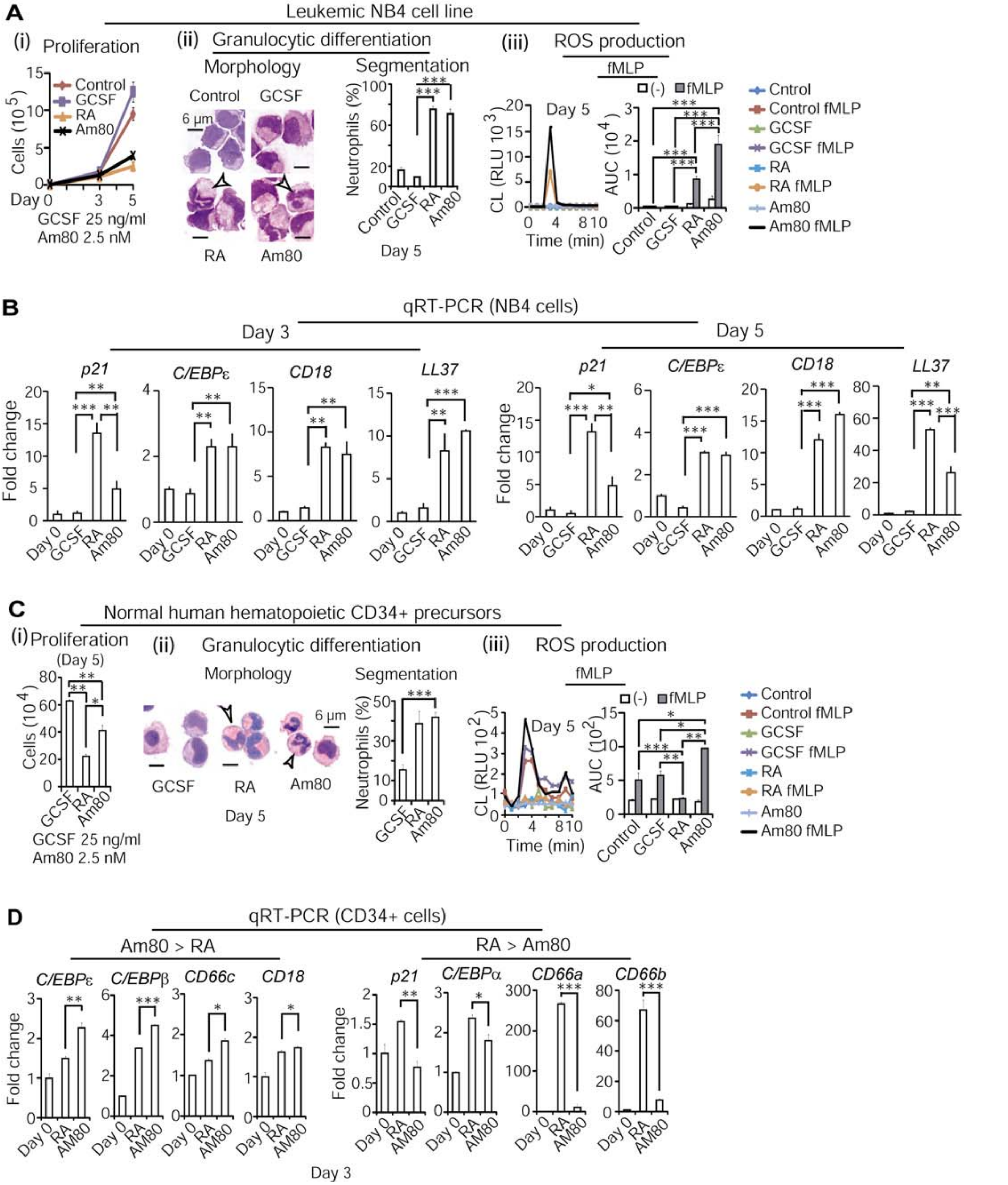

Appendix Figure S1. Am80 promotes neutrophil differentiation, enhances ROS production, and alters transcription of RA-target genes in human hematopoietic precursors. (**A**) Both Am80 and RA, but not GCSF, inhibited leukemic growths of NB4 cells (section *i*) while enhancing granulocytic morphologic differentiation (section *ii*). Am80 promoted significantly higher ROS production than did RA, whereas GCSF failed to induce ROS production (section *iii*). White arrows indicate nuclear segmentations of neutrophils. ROS, reactive oxygen species. CL, chemiluminescence; RLU, relative light units; AUC, area under the curve. Leukemia NB4 cells, as described before (Chaudhry et al, 2012; Wang et al, 2006), were tested for mycoplasma-free by PCR analysis. These NB4 cells from passages 5 to 15 were used for less than five months. (**B**) qRT-PCR analyses of differential expression of RA-target genes mediated by RA, Am80, and GCSF in parallel. RA promoted the highest expression of cell cycle inhibitor $p21^{Cip/Kip}$. (**C**) In normal primary human hematopoietic CD34+ precursors, RA inhibited proliferation (section *i*) and promoted granulocytic differentiation (section *ii*) whereas was much less efficient for inducing ROS production (section *iii*). On the other hand, GCSF displayed the least effect on inducing granulocytic morphology differentiation compared RA or Am80, while Am80 significantly promoted ROS production, compared to GCSF or RA. (**D**) Compared to RA, Am80 altered transcription pattern of RA-induced target genes regulating granulocytic differentiation in CD34+ cells. Data are shown as mean ± SD and represent at least 2 independent experiments with similar results. RA (Sigma-Aldrich) was dissolved in 100% ethanol, and 1 μM RA was used for NB4 cells while 0.5 μM RA for CD34+ cells. *, $P < 0.05$; **, $P < 0.01$; ***, $P < 0.001$. Numbers of P values are provided in Appendix Table S4.

Appendix Figure S2

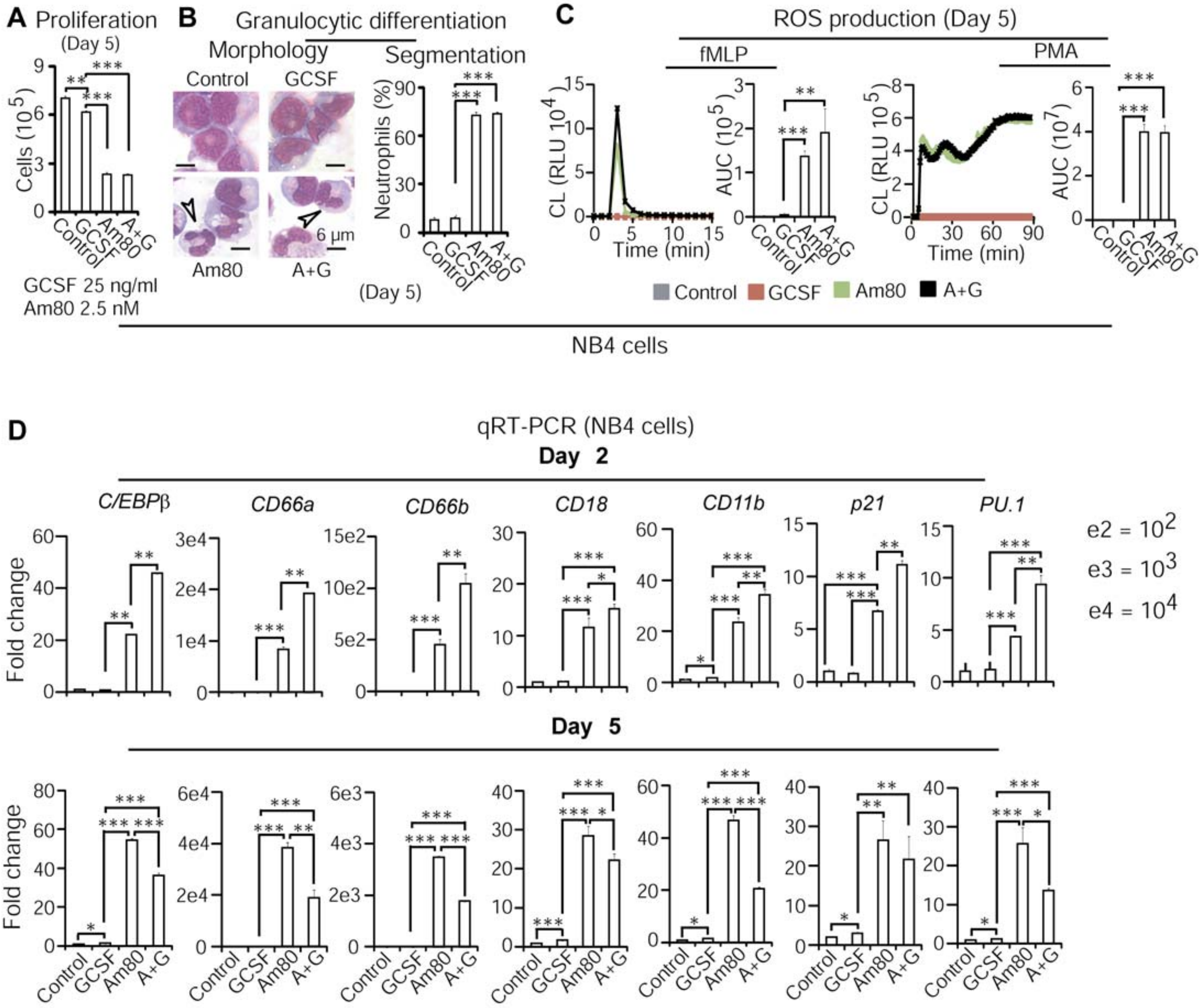

Appendix Figure S2. Am80-GCSF combination significantly promotes granulocytic differentiation, ROS production, and alters transcription of RA-target genes in NB4 cells. (**A-C**) Am80-GCSF combination significantly inhibited cell proliferation (panel A). This inhibition was associated with enhanced granulocytic differentiation (panel B) and promoted ROS production (panel C) on day 5. GCSF failed to induce granulocytic differentiation and ROS production in NB4 cells. White arrows indicate nuclear segmentation of neutrophils. (**D**) Am80-GCSF induced significantly higher expression of RA-target genes on day 2 compared to Am80, whereas Am80 induced higher expression of these same RA-target genes on day 5. GCSF did not induce transcription

of RA-target genes in NB4 cells. Data are shown as mean ± SD and represent at least two independent experiments with similar results. *: $P < 0.05$; **: $P < 0.01$; ***: $P < 0.001$. Numbers of P values are provided in Appendix Table S4.

Appendix Figure S3

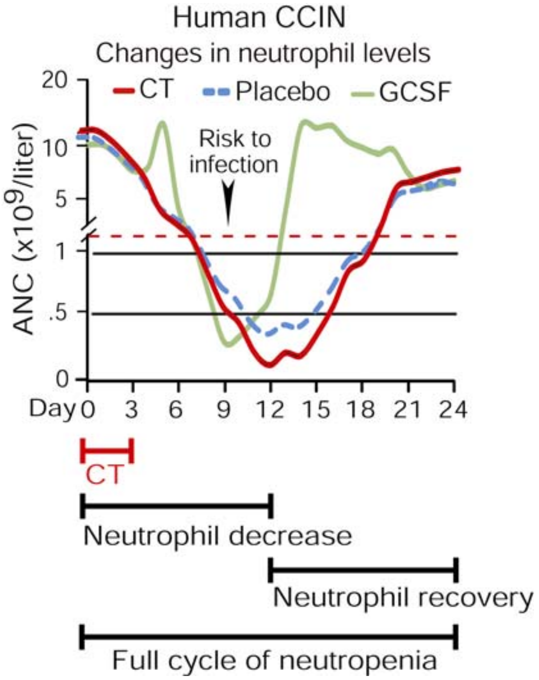

Appendix Figure S3. Neutrophil levels in human cancer chemotherapy-induced neutropenia. Human cancer chemotherapy-induced neutropenia (CCIN) consists of neutrophil decrease and neutrophil recovery stages, and the median duration of CCIN is 9-15 days (ANC <1.5 x 10^9/L), as described by other groups' studies (Crawford et al, 1991; Trillet-Lenoir et al, 1993). GCSF cannot prevent neutrophil reduction in the neutrophil decrease stage of human CCIN. During neutrophil recovery stage, although GCSF profoundly increases the number of neutrophils to reduce the duration of neutropenia, these neutrophils are inadequately differentiated with impaired microbicidal function that cannot effectively reduce human CCIN-associated infection and mortality (Bohlius et al, 2008; Gurion et al, 2012; Hartmann et al, 1997; Heath et al, 2003; Mhaskar et al, 2014; Sung et al, 2007). CT, chemotherapy; ANC, absolute neutrophil count.

Appendix Figure S4

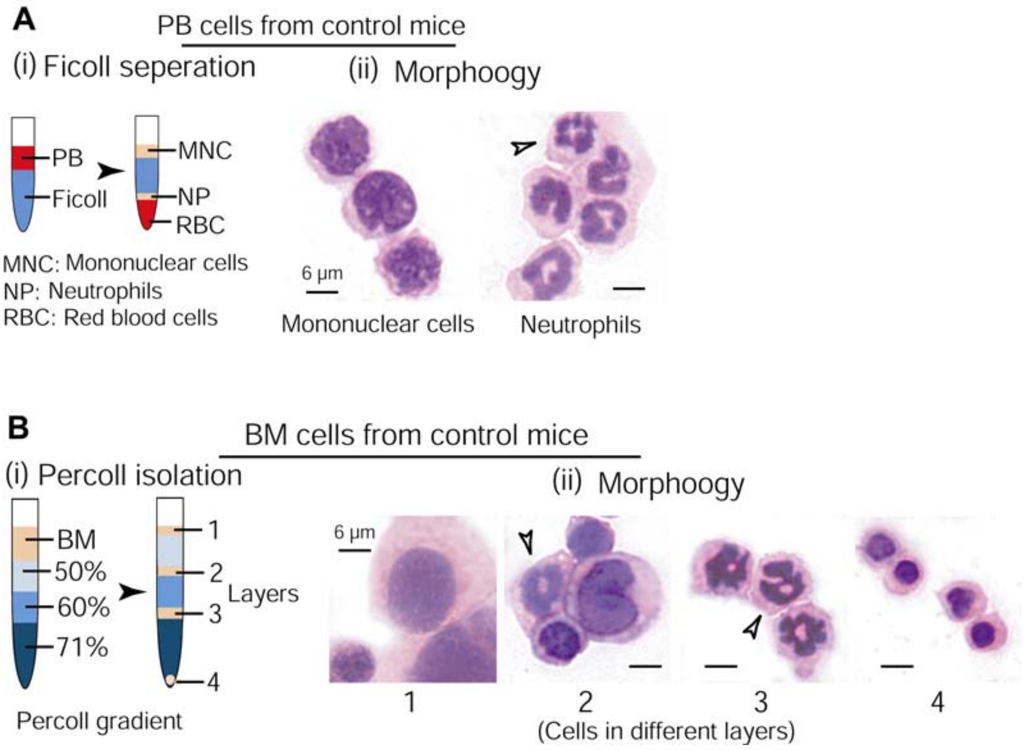

Appendix Figure S4. Identifying PB and BM neutrophils collected from control mice. (**A**) PB mononuclear cells and neutrophils from control mice were separated with Ficoll isolation and identified with granulocytic morphologic analysis. (**B**) Less- and more-matured BM neutrophils of control mice were identified in the second and third layers, respectively, with Percoll gradient segregation and granulocytic morphologic analysis.

Appendix Figure S5

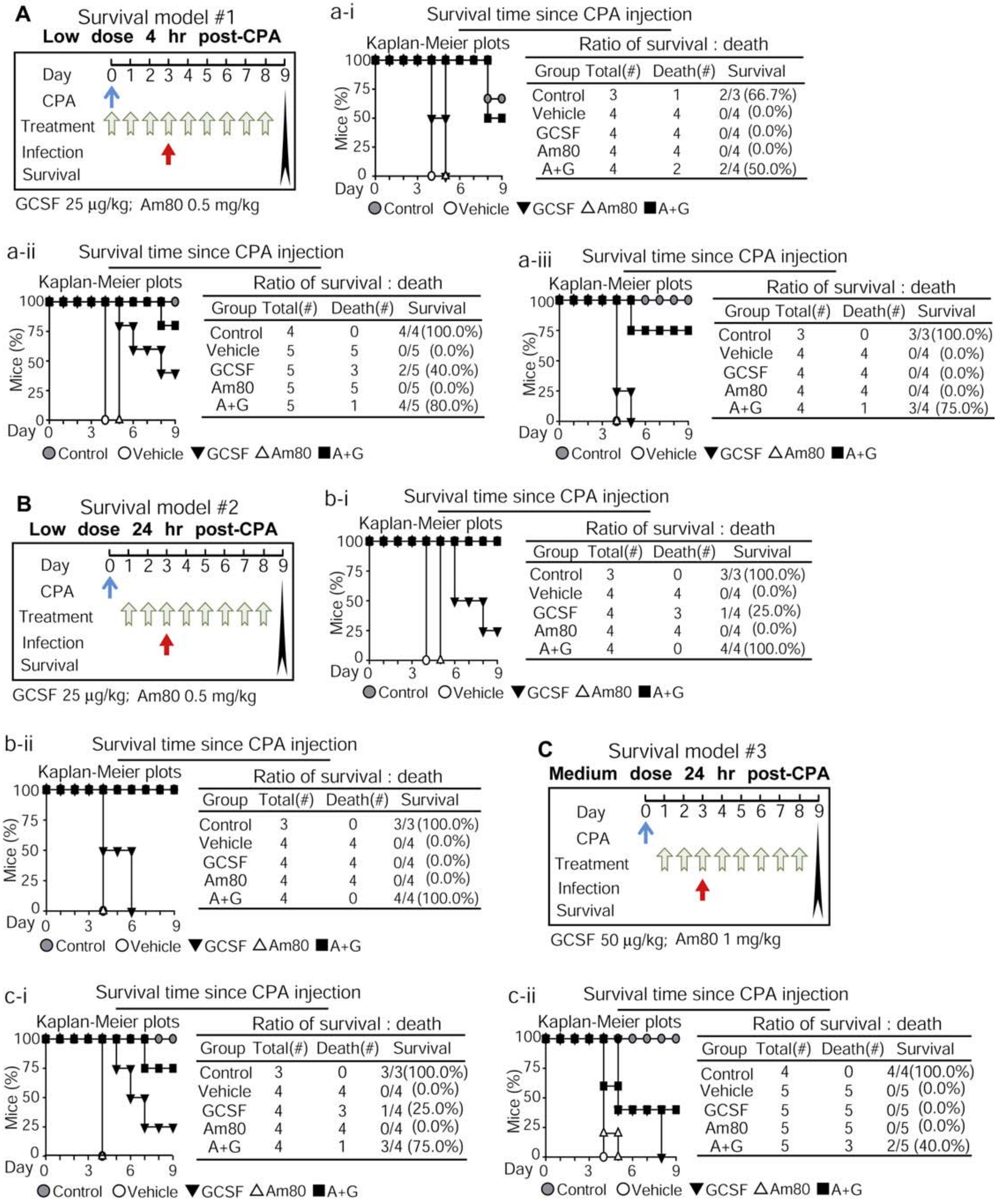

Appendix Figure S5. Each of individual tests performed in different survival models of mouse CCIN. (**A**) Survival model #1 of mouse CCIN treated with low dose of Am80-GCSF after 4 hrs of CPA injection. (a-i to a-iii) Three independent experiments were performed with survival model #1 to evaluate the infection-related mortality of CCIN mice. Kaplan-Meier plots analysis was used to calculate survival rate of CCIN mice. (**B**) Survival model #2 of mouse CCIN treated with low dose of Am80-GCSF after 24 hrs of CPA injection. (b-i & b-ii) Two independent experiments were performed with survival model #2 to evaluate the infection-related mortality of CCIN mice. Kaplan-Meier plots analysis was used to calculate survival rate of CCIN mice. (**C**) Survival model #3 of mouse CCIN treated with medium dose of Am80-GCSF after 24 hrs of CPA injection. (c-i & c-ii) Two independent experiments were performed with survival model #3 to evaluate the infection-related mortality of CCIN mice. Kaplan-Meier plots analysis was used to calculate survival rate of CCIN mice.

2) Appendix Supplemental Tables and Table Legends

Appendix Table S1

A

Am80

Human plasma concentration*

Oral dose (mg/m^2)	PK Cmax (ng/ml)	PK Cmax (nM)
2	18.0	51
4	44.9	128
6	60.9	171

Cmax: Peak plasma concentration

Cell culture concentration

Cell types	Dose (nM)
CD34+	2.5
NB4	2.5
Normal specimen	10.0
AML specimen	20.0, 50.0, 100.0, 150.0

Am80 molecular weight: 351.44 g/mol

Clinical dose of Am80 for APL: 6 to 9 mg/m^2/day

*Investigator's brochure, Tamibarotene (INNO-507)

B

GCSF

Human plasma concentration**

Subcutaneous dose (μg/kg)	PK Cmax (ng/ml)
3.45	4
11.5	49

Cell culture concentration

Cell types	Dose (ng/ml)
CD34+	25
NB4	25
Primary specimen	25

Clinical dose of GCSF for CCIN: 5 to 10 μg/kg/day

**NEUPOGEN® (filgrastim) Prescribing Information www.NEUPOGEN.COM, 1991-2015, Amgen Inc.

Appendix Table S1. Converting human plasma concentrations of GCSF and Am80 to equivalent cell culture doses. Am80 and GCSF doses used in cell culture were derived from their corresponding concentrations in human plasma. **A**) Am80 doses used in cell cultures were within the pharmacokinetics (PK) plasma concentrations determined in both healthy subjects and APL patients. **B**) GCSF dose of 25 ng/ml for *in vitro* inducing granulocytic differentiation of hematopoietic precursors has been well-established (Ding et al, 2013; Hao et al, 1998; Lou et al, 2013; Luo et al, 2007). This GCSF dose established for cell culture is in the medium range of its clinical application.

Appendix Table S2

Gene name	GenBank No.	Forward	Reverse
PU.1	NM_001080547.1	5'-AGCTCAGATGAGGAGGAGGG-3'	5'-AACAGGAACTGGTACAGGCG-3'
C/EBPα	NM_004364.4	5'-GTCGGTGGACAAGAACAGCA-3'	5'-CCTTCTGCTGCGTCTCCACG-3'
C/EBPβ	NM_005194.3	5'-AACTCTCTGCTTCTCCCTCTG-3	5'-AAGCCCGTAGGAACATCTTT-3'
C/EBPε	NM_001805.3	5'-TGGAGAGGCATTCCCAGAAG-3'	5'-GCGACTCCTGGCCTATTCAG-3'
RARβ2	NM_016152.3	5'-AAGTGAGCTGTTCAGAGGCA-3'	5'-AATGCGTTCCGGATCCTACC-3'
CD18	NM_000211.4	5'-TCTGCTTCTGCGGGAAGTG-3'	5'-ATGTACTTGCCACAGGGTGA-3'
CD11b	NM_001145808.1	5'-GCCAGTACTGAGAGTCAAGG-3'	5'-CAACACTCTGGATCTGTCCT-3'
p21 $^{Cip/Kip}$	NM_000389.4	5'-TGCCGAAGTCAGTTCCTTGT-3'	5'-CATTAGCGCATCACAGTCGC-3'
CD66a	NM_001024912.2	5'-AAAATGGCCTCTCACCTGGG-3'	5'-GGGTCATTGGAGTGGTCCTG-3'
CD66b	NM_001816.3	5'-GATCCTATGCCTGCCACACC-3'	5'-GCTGACAGTGGCTCTAGCTG-3'
CD66c	NM_002483.6	5'-TATCCCTGAGAGGAGGCTCA-3'	5'-TCCAGGGGACATGCAATCTG-3'
LL-37	NM_004345.4	5'-GACAGTGACCCTCAACCAG-3'	5'-CCTGGGTACAAGATTCCGCA-3'
GAPDH	NM_002046.5	5'-CATGGCCTCCAAGGAGTAAG-3'	5'-AGGGGTCTACATGGCAACTG-3'

Appendix Table S2. Primers of 12 different RA-target genes used in qRT-PCR analyses.

Appendix Table S3

Control and test groups used in six different mouse CCIN models

Group	Control	Vehicle	GCSF	Am80	A+G
CPA	−	+	+	+	+
Infection	+	+	+	+	+

Appendix Table S3. Control and vehicle groups were in parallel used in different mouse CCIN models for both monitoring and evaluating *in vivo* tests.

Appendix Table S4

The numbers of P values derived from statistical tests

Figure 1	Section		P-value		test performed
1 A	section i	GCSF vs. Am80	0.000507961	***	t-test
		Am80 vs. A+G	0.000361862	***	t-test
	section ii	GCSF vs. AM80	0.049410586	*	t-test
		A+G vs. Am80	0.019511666	*	t-test
		A+G vs. GCSF	0.002320299	**	t-test
	section iii	A+G vs. GCSF	8.75834E-06	***	t-test
		A+G vs. Am80	0.000116081	***	t-test
		GCSF vs. Am80	0.041188173	*	t-test
	section iv				
	fMLP	A+G vs. Am80	2.05126E-08	***	t-test
		A+G vs. GCSF	6.21184E-09	***	t-test
		GCSF vs. AM80	0.000212119	***	t-test
	PMA	A+G vs. GCSF	6.89633E-05	***	t-test
		A+G vs. AM80	5.45779E-05	***	t-test
		AM80 vs. CONTROL	0.003398608	**	t-test
		GCSF vs. CONTROL	0.000603203	***	t-test
1 B		RARB2			
	day-1	CONTROL vs. GCSF	0.002081323	**	t-test
		CONTROL vs. AM80	0.002911097	**	t-test
		GCSF vs. AM80	0.041328424	*	t-test
		GCSF vs. A+G	0.007199199	**	t-test
		AM80 vs. A+G	0.001283229	**	t-test
	day-2	RARB2			
		CONTROL vs. GCSF	0.011796117	*	t-test
		GCSF vs. AM80	0.000214155	***	t-test
		GCSF vs. A+G	0.039904907	*	t-test
		AM80 vs. A+G	0.005037947	**	t-test
	day-6	RARB2			
		CONTROL vs. GCSF	0.001021988	**	t-test
		CONTROL vs. A+G	0.015849947	*	t-test
		GCSF vs. AM80	3.58724E-05	***	t-test
		AM80 vs. A+G	0.00152979	**	t-test
	day-1	C/EBPE			

		CONTROL vs. GCSF	0.000233461	***	t-test
		CONTROL vs. AM80	0.000744687	***	t-test
		GCSF vs. A+G	0.001129485	**	t-test
		AM80 vs. A+G	0.002317695	**	t-test
	day-2	C/EBPE			
		CONTROL vs. GCSF	0.012851219	*	t-test
		GCSF vs. AM80	0.002227852	**	t-test
		GCSF vs. A+G	0.003629911	**	t-test
		AM80 vs. A+G	0.011847646	*	t-test
	day-6	C/EBPE			
		CONTROL vs. GCSF	0.020892577	*	t-test
		CONTROL vs. A+G	0.006056347	**	t-test
		GCSF vs. AM80	0.012460429	*	t-test
		AM80 vs. A+G	0.003750998	**	t-test
	day-1	CD66C			
		CONTROL vs. GCSF	0.002202712	**	t-test
		CONTROL vs. AM80	0.002403684	**	t-test
		GCSF vs. A+G	0.01196121	*	t-test
		AM80 vs. A+G	0.002873151	**	t-test
	day-2	CD66C			
		GCSF vs. AM80	0.000156977	***	t-test
		GCSF vs. A+G	0.001040212	**	t-test
		AM80 vs. A+G	0.000699928	***	t-test
	day-6	CD66C			
		CONTROL vs. GCSF	0.000810754	***	t-test
		CONTROL vs. AM80	5.04839E-05	***	t-test
		CONTROL vs. A+G	0.039969056	*	t-test
		GCSF vs. AM80	2.76114E-06	***	t-test
		GCSF vs. A+G	0.015221519	*	t-test
		AM80 vs. A+G	0.000339287	***	t-test
	day-1	CD66B			
		CONTROL vs. A+G	0.029790985	*	t-test
		GCSF vs. A+G	0.009658543	**	t-test
		AM80 vs. A+G	0.033069002	*	t-test
	day-2	CD11B			
		CONTROL vs. AM80	0.005083568	**	t-test
		GCSF vs. AM80	0.01498158	*	t-test
		GCSF vs. A+G	0.029502637	*	t-test

	day-6	CD18			
		CONTROL vs. GCSF	0.000131892	***	t-test
		CONTROL vs. AM80	0.030181164	*	t-test
		CONTROL vs. A+G	0.004741731	**	t-test
		GCSF vs. AM80	0.00264742	**	t-test
		GCSF vs. A+G	0.000283134	***	t-test
		AM80 vs. A+G	0.001843599	**	t-test
1 C	section ii	CONTROL vs. GCSF	0.000708599	***	t-test
		CONTROL vs. AM80	0.000332137	***	t-test
		CONTROL vs. A+G	0.000181248	***	t-test
		GCSF vs. AM80	0.000537741	***	t-test
		GCSF vs. A+G	0.000227347	***	t-test
		AM80 vs. A+G	0.000523553	***	t-test
1 D	section i	neutrophil vs. (-)	1.96E-15	***	t-test
	section ii	AM80 vs. CONTROL	1.22969E-07	***	t-test
		A+G vs. CONTROL	5.68111E-06	***	t-test
		A+G VS. GCSF	8.35227E-06	***	t-test
	section iii	A+G vs. GCSF	9.298E-07	***	t-test
		A+G vs. AM80	9.62803E-06	***	t-test
		AM80 vs. CONTROL	0.025814088	*	t-test
1 E		CONTROL vs. CD18 Ab	2.22866E-06	***	t-test
		CONTROL vs. CD11b Ab	4.56436E-06	***	t-test
		CONTROL vs. COMB	6.13816E-05	***	t-test
1 F	section i	GCSF vs. AM80	0.000686193	***	t-test
		GCSF vs. A+G	4.05964E-07	***	t-test
		AM80 vs. A+G	0.00786187	**	t-test
	section ii	GCSF vs. AM80	6.16266E-06	***	t-test
		GCSF vs. A+G	1.09423E-05	***	t-test
		AM80 vs. AM80+CD18	0.012608771	*	t-test
		A+G vs. A+G+CD18	2.51628E-05	***	t-test

Figure 2	Section		P-value		test performed
2 A	section ii				
	FMLP	A+G vs. GCSF	5.1836E-06	***	t-test
		A+G vs. AM80	2.18769E-05	***	t-test
		AM80 vs. CONTROL	0.030218647	*	t-test
	PMA	A+G vs. GCSF	0.003720838	**	t-test
		A+G vs. AM80	1.51091E-07	***	t-test
		GCSF vs. AM80	2.23123E-07	***	t-test
2 B	section i				
	Day-2	RARB2			
		GCSF vs. A+G	0.000215334	***	t-test
		GCSF vs. AM80	0.000147551	***	t-test
		AM80 vs. A+G	0.010643082	*	t-test
	Day-3	RARB2			
		AM80 vs. A+G	0.04581776	*	t-test
		GCSF vs. A+G	1.81038E-06	***	t-test
		GCSF vs. AM80	0.001573663	**	t-test
	Day-6	RARB2			
		CONTROL vs. A+G	0.006137164	**	t-test
		AM80 vs. A+G	0.001765861	**	t-test
		GCSF vs. A+G	0.000412182	***	t-test
	Day-2	C/EBPE			
		CONTROL vs. AM80	3.79162E-05	***	t-test
		AM80 vs. A+G	0.00547171	**	t-test
		GCSF vs. A+G	0.000163628	***	t-test
		GCSF vs. AM80	0.000653074	***	t-test
	Day-3	C/EBPE			
		AM80 vs. A+G	0.009298449	**	t-test
		GCSF vs. A+G	0.045894711	*	t-test
	Day-6	C/EBPE			
		CONTROL vs. A+G	0.000622879	***	t-test
		AM80 vs. A+G	0.000360051	***	t-test
		GCSF vs. A+G	7.99109E-05	***	t-test
	Day-2	CD11B			
		CONTROL vs. AM80	0.000114921	***	t-test
		GCSF vs. A+G	0.000597698	***	t-test

		GCSF vs. AM80	0.000112611	***	t-test
	Day-3	CD11B			
		CONTROL vs. A+G	0.023759931	*	t-test
		AM80 vs. A+G	0.035363674	*	t-test
		GCSF vs. AM80	0.002787172	**	t-test
	Day-6	CD11B			
		AM80 vs. A+G	0.037309836	*	t-test
		GCSF vs. A+G	0.021684306	*	t-test
	section ii	CONTROL vs. GCSF	0.005948397	**	t-test
		CONTROL vs. A+G	0.007461105	**	t-test
	section iii				
	FMLP	AM80 vs. A+G	0.010685223	*	t-test
	PMA	AM80 vs. A+G	0.015180143	*	t-test

Figure 3	Section		P-value		test performed
3 A	section i	CONTROL vs. GCSF	0.015405547	*	t-test
		GCSF vs. Am80	0.008523155	**	t-test
		GCSF vs. A+G	0.001211221	**	t-test
		Am80 vs. A+G	0.009552252	**	t-test
	section ii	GCSF vs. A20	0.000433008	***	t-test
3 B	section i	CONTROL vs. GCSF	0.004445595	**	t-test
		GCSF vs. A20	7.70145E-05	***	t-test
		GCSF vs. A20+G	0.043395245	*	t-test
		GCSF vs. A100+G	0.008254467	**	t-test
		GCSF vs. A150+G	9.57225E-05	***	t-test
		A20 vs. A100	0.000199269	***	t-test
	section ii	CONTROL vs. GCSF	0.000151011	***	t-test
		GCSF vs. A20	1.30013E-05	***	t-test
		GCSF vs. A20+G	3.97667E-06	***	t-test
		GCSF vs. A100+G	9.57445E-07	***	t-test
		GCSF vs. A150+G	4.9421E-07	***	t-test
		A20 vs. A20+G	0.001826261	**	t-test

	section iii	CONTROL vs. GCSF	0.003480947	**	t-test
		GCSF vs. A20	0.001089883	**	t-test
		GCSF vs. A20+G	0.017912804	*	t-test
		GCSF vs. A100+G	0.006184543	**	t-test
		GCSF vs. A150+G	0.010636628	*	t-test
		A100 vs. A20+G	3.57879E-05	***	t-test
	section iv	CONTROL vs. GCSF	0.000184087	***	t-test
		GCSF vs. A20	3.41902E-05	***	t-test
		GCSF vs. A100+G	2.06111E-05	***	t-test
		GCSF vs. A150+G	1.38125E-05	***	t-test
		A20 vs. A20+G	1.9079E-05	***	t-test
3 C	section i				
	proliferation	CONTROL vs. GCSF	0.00014365	***	t-test
		GCSF vs. A150	0.004845773	**	t-test
		GCSF vs. A150+G	0.001760537	**	t-test
		A150 vs. A150+G	0.049190089	*	t-test
	killing	A+G vs. CONTROL	9.80927E-09	***	t-test
		A+G vs. GCSF	5.59929E-08	***	t-test
		A+G vs. AM80	4.93609E-06	***	t-test
		GCSF vs. CONTROL	0.00149551	**	t-test
		AM80 vs. CONTROL	0.000168509	***	t-test
	section ii				
	proliferation	CONTROL vs. A150	4.89309E-06	***	t-test
		CONTROL vs. A150+G	3.43708E-06	***	t-test
		GCSF vs. A150	3.28472E-06	***	t-test
		GCSF vs. A150+G	2.1476E-06	***	t-test
	killing	A+G vs. CONTROL	3.55759E-07	***	t-test
		A+G vs. GCSF	1.02233E-06	***	t-test
		A+G vs. AM80	1.95726E-09	***	t-test
		AM80 vs. CONTROL	0.021403248	*	t-test
3 D	section i	CONTROL vs. GCSF	0.000142006	***	t-test
		CONTROL vs. A150	0.000435875	***	t-test
		CONTROL vs. A150+G	0.000341065	***	t-test
		GCSF vs. A150	2.94998E-05	***	t-test
		GCSF vs. A150+G	2.08592E-05	***	t-test
		A150 vs. A150+G	0.026997302	*	t-test

	section ii	CONTROL vs. GCSF	0.000118898	***	t-test
		CONTROL vs. A150	1.76779E-06	***	t-test
		GCSF vs. A150	1.24454E-06	***	t-test
		GCSF vs. A150+G	9.79659E-05	***	t-test
		A150 vs. A150+G	2.23732E-07	***	t-test
	section iii	A+G vs. GCSF	1.38666E-05	***	t-test
		A+G vs. AM80	0.000132157	***	t-test
		GCSF vs. CONTROL	0.00586509	**	t-test
		AM80 vs. CONTROL	0.003781889	**	t-test
	section iv	CONTROL vs. A150	0.000679624	***	t-test
		CONTROL vs. A150+G	1.16316E-05	***	t-test
		GCSF vs. A150	0.00084841	***	t-test
		GCSF vs. A150+G	1.36003E-05	***	t-test
		A150 vs. A150+G	0.00466378	**	t-test
	section v	RARb2			
		A150 vs. CONTROL	0.000785505	***	t-test
		A150 vs. GCSF	0.001965128	**	t-test
		A150+G vs. A150	3.84172E-05	***	t-test
		CEBPe			
		GCSF vs. CONTROL	0.007041532	**	t-test
		A150 vs. GCSF	6.2234E-07	***	t-test
		A150+G vs. A150	0.000405437	***	t-test
		CD66c			
		GCSF vs. CONTROL	0.000288632	***	t-test
		A150 vs. GCSF	0.002612316	**	t-test
		A150+G vs. A150	0.001023201	**	t-test

**Figure 4**	**Section**		**P-value**		**test performed**
4 E	section i	GCSF vs. AM80	0.000101111	***	t-test
	section ii	GCSF vs. AM80	0.000371622	***	t-test
4 F	section i	CONTROL vs. VEHICLE	0.039311426	*	t-test
		VEHICLE vs. GCSF	0.029330023	*	t-test
		VEHICLE vs. AM80	0.008443809	**	t-test
		GCSF vs. AM80	0.027010983	*	t-test
		GCSF vs. A+G	0.001746488	**	t-test

		AM80 vs. A+G	0.000107685	***	t-test
	section ii	CONTROL vs. VEHICLE	0.019103662	*	t-test
		CONTROL vs. GCSF	0.000997507	***	t-test
		VEHICLE vs. AM80	0.027123892	*	t-test
		GCSF vs. AM80	0.006343464	**	t-test
		GCSF vs. A+G	0.01559614	*	t-test
		AM80 vs. A+G	0.026967131	*	t-test
	section iii	CONTROL vs. VEHICLE	0.011121655	*	t-test
		VEHICLE vs. AM80	0.03022058	*	t-test
		GCSF vs. AM80	0.015407895	*	t-test
		AM80 vs. A+G	0.005188128	**	t-test
4 I	section i	CONTROL vs. GCSF	0.000379795	***	t-test
		VEHICLE vs. GCSF	2.34434E-05	***	t-test
		GCSF vs. AM80	0.002684044	**	t-test
		GCSF vs. A+G	0.000403473	***	t-test
	section ii	CONTROL vs. GCSF	1.38267E-07	***	t-test
		VEHICLE vs. GCSF	0.001066013	**	t-test
		GCSF vs. AM80	7.65501E-05	***	t-test
		GCSF vs. A+G	0.036176375	*	t-test
4 J	section i	CONTROL vs. VEHICLE	2.88423E-06	***	t-test
		CONTROL vs. A+G	0.018249442	*	t-test
		VEHICLE vs. AM80	2.36543E-05	***	t-test
		VEHICLE vs. A+G	0.00130131	**	t-test
		GCSF vs. AM80	0.017353396	*	t-test
	section ii	CONTROL vs. VEHICLE	0.049542753	*	t-test
		CONTROL vs. GCSF	0.009862624	**	t-test
		VEHICLE vs. GCSF	0.025986032	*	t-test
		VEHICLE vs. AM80	0.031824836	*	t-test
		GCSF vs. AM80	0.009180151	**	t-test
	section iii	CONTROL vs. AM80	0.017553084	*	t-test
		VEHICLE vs. GCSF	0.015309031	*	t-test
		VEHICLE vs. A+G	0.028345175	*	t-test
		GCSF vs. AM80	9.61344E-05	***	t-test
		AM80 vs. A+G	0.000409851	***	t-test

Figure 5	Section		P-value		test performed
5 C	section i	CONTROL vs. GCSF	6.89177E-05	***	t-test
		VEHICLE vs. GCSF	0.010551546	*	t-test
		VEHICLE vs. AM80	0.002569943	**	t-test
		VEHICLE vs. A+G	0.000702996	***	t-test
		GCSF vs. A+G	0.003262141	**	t-test
	section ii	CONTROL vs. VEHICLE	0.001081923	**	t-test
		VEHICLE vs. GCSF	0.002335775	**	t-test
		VEHICLE vs. AM80	0.001832213	**	t-test
		GCSF vs. A+G	0.00022431	***	t-test
		AM80 vs. A+G	9.30121E-05	***	t-test
5 D	Second layer	GCSF vs. AM80	0.001670886	**	t-test
		AM80 VS. A+G	7.64088E-07	***	t-test
	Third layer	GCSF VS. AM80	0.00033732	***	t-test
		AM80 vs. A+G	0.008661791	**	t-test
5 E	mononuclear layer	CONTROL vs. A+G	0.000172534	***	t-test
		VEHICLE vs. A+G	0.000543776	***	t-test
		GCSF vs. A+G	0.000832008	***	t-test
		AM80 vs. A+G	1.01936E-05	***	t-test
	neutrophil layer	CONTROL vs. VEHICLE	0.000258254	***	t-test
		CONTROL vs. GCSF	0.000335171	***	t-test
		CONTROL vs. AM80	7.26645E-05	***	t-test
		CONTROL vs. A+G	0.000119618	***	t-test
		GCSF vs. AM80	0.00027174	***	t-test
		AM80 vs. A+G	0.00018292	***	t-test

Figure 6	Section		P-value		test performed
6 B	section i	Am80 + GCSF vs. GCSF	0.002	**	Log Rank (Mantel-Cox) test
6 E	section i	Am80 + GCSF vs. GCSF	0.001	**	Log Rank (Mantel-Cox) test
6 G	section i	Am80 + GCSF vs. GCSF	0.128		Log Rank (Mantel-Cox) test

Figure 7	Section		P-value		test performed
7 A	second layer	CONTROL vs. GCSF	0.002069261	**	t-test
		CONTROL vs. A+G	0.01912119	*	t-test
	third layer	CONTROL vs. A+G	1.47116E-07	***	t-test
		GCSF vs. A+G	3.60129E-05	***	t-test
7 B	mononuclear layer	CONTROL vs. GCSF	0.000211413	***	t-test
		CONTROL vs. A+G	0.003623241	**	t-test
	neutrophil layer	CONTROL vs. GCSF	0.000937776	***	t-test
		CONTROL vs. A+G	0.000358809	***	t-test
7 C	section i				
	mononuclear layer	CONTROL vs. A+G	0.004621978	**	t-test
	section iv	CONTROL vs. GCSF	0.037916073	*	t-test
		CONTROL vs. A+G	0.013570868	*	t-test
7 D		CONTROL vs. GCSF	0.029995596	*	t-test
7 E	section ii	CONTROL vs. GCSF	0.007958731	**	t-test
		GCSF vs. A+G	0.008884621	**	t-test
	section iii	CONTROL vs. GCSF	0.006175169	**	t-test
		GCSF vs. A+G	0.006175169	**	t-test

Appendix Fig. S1	Section		P-value		test performed
S1A	section ii	GCSF vs. RA	6.43244E-05	***	t-test
		GCSF vs. AM80	0.00047447	***	t-test
	section iii	AM80+FMLP vs. CONTROL+FMLP	3.05061E-05	***	t-test
		AM80+FMLP vs. GCSF+FMLP	3.05274E-05	***	t-test
		AM80+FMLP vs. RA+FMLP	0.000271653	***	t-test
		RA+FMLP vs. CONTROL+FMLP	1.03733E-05	***	t-test
		RA+FMLP vs. GCSF+FMLP	1.04376E-05	***	t-test

S1B	DAY-3	P21			
		GCSF vs. RA	0.000170952	***	t-test
		GCSF vs. AM80	0.006106507	**	t-test
		RA vs. AM80	0.001591935	**	t-test
	DAY-3	CEBPE			
		GCSF vs. RA	0.003006197	**	t-test
		GCSF vs. AM80	0.008455783	**	t-test
	DAY-3	CD18			
		GCSF vs. RA	0.002474417	**	t-test
		GCSF vs. AM80	0.00964762	**	t-test
	DAY-3	LL37			
		GCSF vs. RA	0.004623589	**	t-test
		GCSF vs. AM80	0.000146811	***	t-test
	DAY-5	P21			
		GCSF vs. RA	7.4474E-05	***	t-test
		GCSF vs. AM80	0.011420255	*	t-test
		RA vs. AM80	0.005706583	**	t-test
	DAY-5	CEBPE			
		GCSF vs. RA	3.43255E-05	***	t-test
		GCSF vs. AM80	9.83852E-05	***	t-test
	DAY-5	CD18			
		GCSF vs. RA	0.000249872	***	t-test
		GCSF vs. AM80	1.91469E-05	***	t-test
	DAY-5	LL37			
		GCSF vs. RA	6.112E-08	***	t-test
		GCSF vs. AM80	0.001109431	**	t-test
		RA vs. AM80	0.000948947	***	t-test
S1C	section i	GCSF vs. AM80	0.009589075	**	t-test
		GCSF vs. RA	0.003031728	**	t-test
		RA vs. AM80	0.018691415	*	t-test
	section ii	AM80 vs. GCSF	1.4525E-06	***	t-test
	section iii	AM80+FMLP vs. CONTROL+FMLP	0.013141707	*	t-test
		AM80+FMLP vs. GCSF+FMLP	0.030642709	*	t-test
		AM80+FMLP vs. RA+FMLP	0.004618911	**	t-test

		RA+FMLP vs. CONTROL+FMLP	0.000932011	***	t-test
		RA+FMLP vs. GCSF+FMLP	0.001899765	**	t-test
S1D		RA vs. AM80			
		CEBPE	0.003856126	**	t-test
		CEBPB	0.000769532	***	t-test
		CD66C	0.017367763	*	t-test
		CD18	0.048213791	*	t-test
		P21	0.002174544	**	t-test
		CEBPA	0.011533013	*	t-test
		CD66A	1.6E-05	***	t-test
		CD66B	0.000503848	***	t-test

**Appendix Fig. S2**	**Section**		**P-value**		**test performed**
S2A		CONTROL vs. GCSF	0.001193974	**	t-test
		GCSF vs. A2.5	3.29739E-06	***	t-test
		GCSF vs. A+G	5.02668E-07	***	t-test
S2B		GCSF vs. AM80	1.24757E-06	***	t-test
		A+G vs. GCSF	9.33657E-08	***	t-test
S2C	FMLP	GCSF vs. AM80	5.91073E-05	***	t-test
		GCSF vs. A+G	0.004395386	**	t-test
	PMA	GCSF vs. AM80	4.1466E-05	***	t-test
		GCSF vs. A+G	3.08968E-05	***	t-test
S2D	DAY-2	CEBPB			
		GCSF vs. AM80	0.004153184	**	t-test
		AM80 vs. A+G	0.003568121	**	t-test
	DAY-5	CEBPB			
		CONTROL vs. GCSF	0.01175814	*	t-test
		GCSF vs. AM80	7.13811E-08	***	t-test
		GCSF vs. A+G	2.86989E-05	***	t-test
		AM80 vs. A+G	0.000454155	***	t-test
	DAY-2	CD66A			

		GCSF vs. AM80	0.000101633	***	t-test
		AM80 vs. A+G	0.001362961	**	t-test
	DAY-5	CD66A			
		GCSF vs. AM80	4.692E-05	***	t-test
		GCSF vs. A+G	0.000474794	***	t-test
		AM80 vs. A+G	0.004581575	**	t-test
	DAY-2	CD66B			
		GCSF vs. AM80	0.000105465	***	t-test
		AM80 vs. A+G	0.00278499	**	t-test
	DAY-5	CD66B			
		GCSF vs. AM80	4.94895E-07	***	t-test
		GCSF vs. A+G	2.99389E-10	***	t-test
		AM80 vs. A+G	0.000279078	***	t-test
	DAY-2	CD18			
		GCSF vs. AM80	0.000673476	***	t-test
		GCSF vs. A+G	2.00585E-05	***	t-test
		AM80 vs. A+G	0.045320536	*	t-test
	DAY-5	CD18			
		CONTROL vs. GCSF	5.19123E-05	***	t-test
		GCSF vs. AM80	5.28823E-05	***	t-test
		GCSF vs. A+G	2.21314E-05	***	t-test
		AM80 vs. A+G	0.023360398	*	t-test
	DAY-2	CD11B			
		CONTROL vs. GCSF	0.025392284	*	t-test
		GCSF vs. AM80	3.0468E-05	***	t-test
		GCSF vs. A+G	1.72488E-05	***	t-test
		AM80 vs. A+G	0.003173738	**	t-test
	DAY-5	CD11B			
		CONTROL vs. GCSF	0.044605425	*	t-test
		GCSF vs. AM80	5.93115E-05	***	t-test
		GCSF vs. A+G	2.84708E-05	***	t-test
		AM80 vs. A+G	1.83442E-05	***	t-test
	DAY-2	P21			
		CONTROL vs. AM80	0.000962673	***	t-test
		GCSF vs. AM80	1.85666E-05	***	t-test
		AM80 vs. A+G	0.004574848	**	t-test

	DAY-5	P21			
		CONTROL vs. GCSF	0.023031408	*	t-test
		GCSF vs. AM80	0.00297427	**	t-test
		GCSF vs. A+G	0.005083563	**	t-test
	DAY-2	PU.1			
		GCSF vs. AM80	2.86118E-05	***	t-test
		GCSF vs. A+G	9.41616E-05	***	t-test
		AM80 vs. A+G	0.004926082	**	t-test
	DAY-5	PU.1			
		CONTROL vs. GCSF	0.026610557	*	t-test
		GCSF vs. AM80	0.000495189	***	t-test
		GCSF vs. A+G	2.64418E-05	***	t-test
		AM80 vs. A+G	0.029140581	*	t-test

3) References

Bohlius J, Herbst C, Reiser M, Schwarzer G, Engert A (2008) Granulopoiesis-stimulating factors to prevent adverse effects in the treatment of malignant lymphoma. *Cochrane Database Syst Rev*(4): CD003189

Chaudhry P, Yang X, Wagner M, Jong A, Wu L (2012) Retinoid-regulated FGF8f secretion by osteoblasts bypasses retinoid stimuli to mediate granulocytic differentiation of myeloid leukemia cells. *Mol Cancer Ther* 11(2): 267-276

Crawford J, Ozer H, Stoller R, Johnson D, Lyman G, Tabbara I, Kris M, Grous J, Picozzi V, Rausch G, et al. (1991) Reduction by granulocyte colony-stimulating factor of fever and neutropenia induced by chemotherapy in patients with small-cell lung cancer. *N Engl J Med* 325(3): 164-170

Ding W, Shimada H, Li L, Mittal R, Zhang X, Shudo K, He Q, Prasadarao NV, Wu L (2013) Retinoid agonist Am80-enhanced neutrophil bactericidal activity arising from granulopoiesis in vitro and in a neutropenic mouse model. *Blood* 121(6): 996-1007

Gurion R, Belnik-Plitman Y, Gafter-Gvili A, Paul M, Vidal L, Ben-Bassat I, Shpilberg O, Raanani P (2012) Colony-stimulating factors for prevention and treatment of infectious complications in patients with acute myelogenous leukemia. *Cochrane Database Syst Rev* 6: CD008238

Hao QL, Smogorzewska EM, Barsky LW, Crooks GM (1998) In vitro identification of single CD34+CD38- cells with both lymphoid and myeloid potential. *Blood* 91(11): 4145-4151

Hartmann LC, Tschetter LK, Habermann TM, Ebbert LP, Johnson PS, Mailliard JA, Levitt R, Suman VJ, Witzig TE, Wieand HS, Miller LL, Moertel CG (1997) Granulocyte colony-stimulating factor in severe chemotherapy-induced afebrile neutropenia. *N Engl J Med* 336(25): 1776-1780

Heath JA, Steinherz PG, Altman A, Sather H, Jhanwar S, Halpern S, Pieters R, Shah N, Steinherz L, Tannous R, Terry W, Trigg ME (2003) Human granulocyte colony-stimulating factor in children with high-risk acute lymphoblastic leukemia: a Children's Cancer Group Study. *J Clin Oncol* 21(8): 1612-1617

Lou S, Liu G, Shimada H, Yang X, He Q, Wu L (2013) The lost intrinsic fragmentation of MAT1 protein during granulopoiesis promotes the growth and metastasis of leukemic myeloblasts. *Stem Cells* 31(9): 1942-1953

Luo P, Wang A, Payne KJ, Peng H, Wang JG, Parrish YK, Rogerio JW, Triche TJ, He Q, Wu L (2007) Intrinsic retinoic acid receptor alpha-cyclin-dependent kinase-activating kinase signaling involves coordination of the restricted proliferation and granulocytic differentiation of human hematopoietic stem cells. *Stem Cells* 25(10): 2628-2637

Mhaskar R, Clark OA, Lyman G, Engel Ayer Botrel T, Morganti Paladini L, Djulbegovic B (2014) Colony-stimulating factors for chemotherapy-induced febrile neutropenia. *Cochrane Database Syst Rev* 10: CD003039

Sung L, Nathan PC, Alibhai SM, Tomlinson GA, Beyene J (2007) Meta-analysis: effect of prophylactic hematopoietic colony-stimulating factors on mortality and outcomes of infection. *Ann Intern Med* 147(6): 400-411

Trillet-Lenoir V, Green J, Manegold C, Von Pawel J, Gatzemeier U, Lebeau B, Depierre A, Johnson P, Decoster G, Tomita D, et al. (1993) Recombinant granulocyte colony stimulating factor reduces the infectious complications of cytotoxic chemotherapy. *Eur J Cancer* 29A(3): 319-324

Wang JG, Barsky LW, Davicioni E, Weinberg KI, Triche TJ, Zhang XK, Wu L (2006) Retinoic acid induces leukemia cell G1 arrest and transition into differentiation by inhibiting cyclin-dependent kinase-activating kinase binding and phosphorylation of PML/RARalpha. *FASEB J* 20(12): 2142-2144
